# Characteristics and outcomes of patients with *RET*-fusion positive non-small lung cancer in real-world practice in the United States

**DOI:** 10.1186/s12885-020-07714-3

**Published:** 2021-01-05

**Authors:** Lisa M. Hess, Yimei Han, Yajun Emily Zhu, Naleen Raj Bhandari, Anthony Sireci

**Affiliations:** 1 Eli Lilly and Company, Indianapolis, IN 46254 USA; 2 Loxo Oncology at Lilly, a wholly owned subsidiary of Eli Lilly and Company, Stamford, CT USA

**Keywords:** *RET* fusions, Real-world data, Observational study, Lung cancer, Survival, Tumor response, Retrospective analysis

## Abstract

**Background:**

Contradictory and limited data are available about the presentation and outcomes of patients with *RET*-fusion positive metastatic NSCLC as compared to patients without *RET* fusions. This observational study utilizing a linked electronic health records (EHR) database to genomics testing results was designed to compare characteristics, tumor response, progression-free (PFS) and overall survival (OS) outcomes by *RET* fusion status among patients with metastatic NSCLC treated with standard therapies.

**Methods:**

Adult patients with metastatic NSCLC with linked EHR and genomics data were eligible who received systemic anti-cancer therapy on or after January 1, 2011. Adjusted, using all available baseline covariates, and unadjusted analyses were conducted to compare tumor response, PFS and OS between patients with *RET-*fusion positive and *RET*-fusion negative disease as detected by next-generation sequencing. Tumor response outcomes were analysed using Fisher’s exact test, and time-to-event analyses were conducted using Cox proportional hazards model.

**Results:**

There were 5807 eligible patients identified (*RET*+ cohort, *N* = 46; *RET*- cohort, *N* = 5761). Patients with *RET* fusions were younger, more likely to have non-squamous disease and be non-smokers and had better performance status (all *p* < 0.01). In unadjusted analyses, there were no significant differences in tumor response (*p* = 0.17) or PFS (*p* = 0.06) but OS was significantly different by *RET* status (hazard ratio, HR = 1.91, 95% CI:1.22–3.0, *p* = 0.005). There were no statistically significant differences by *RET* fusion status in adjusted analyses of either PFS or OS (PFS HR = 1.24, 95% CI:0.86–1.78, *p* = 0.25; OS HR = 1.52, 95% CI: 0.95–2.43, *p* = 0.08).

**Conclusions:**

Patients with *RET* fusions have different baseline characteristics that contribute to favorable OS in unadjusted analysis. However, after adjusting for baseline covariates, there were no significant differences in either OS or PFS by *RET* status among patients treated with standard therapy prior to the availability of selective *RET* inhibitors.

## Background

The treatment of patients diagnosed with metastatic non-small cell lung cancer (NSCLC) is increasingly determined by the presence or absence of actionable biomarkers. This is a change that has occurred since 2009, when the first differentiation in treatment was based on tumor histology [1]. Since then, therapies have been approved by regulatory bodies across the world that target epidermal growth factor receptor (EGFR), ROS proto-oncogene 1 (ROS1), v-raf murine sarcoma viral oncogene homolog B (BRAF), anaplastic lymphoma kinase (ALK), mesenchymal-epithelial transition (MET) exon 14, neurootrophic receptor tyrosine kinase (NTRK) and most recently, rearranged during transfection (RET) [2–16].

Selpercatinib was approved for use in the U.S. in May 2020 for patients with *RET*-fusion positive non-small cell lung cancer, *RET*-fusion positive thyroid cancer, or *RET*-mutation positive meduallary thyroid cancer. This approval was based on a single-arm phase I/II trial, LIBRETTO-001, which demonstrated overall tumor response rates of 85 and 64% for patients with RET-fusion positive NSCLC who were treatment naïve and previously treated, respectively [17, 18]. Response rates were similar for patients with *RET* altered thyroid (treatment naïve, 100%; previously treated, 79%) and medullary thyroid cancers (no prior multikinase inhibitors [MKIs], 73%, previously treated with MKIs, 69%) [17]. The characterization of real-world demographics and outcomes in the setting of EGFR, ROS1, BRAF or ALK alterations been investigated in multiple large cohort studies in NSCLC [19–23]. In the case of *RET* fusions, however, less is known. Contradictory and limited data are available about the presentation and outcomes of patients with *RET* fusion-positive metastatic NSCLC.

Many studies of patients with *RET* fusions focus on or include many with early stage disease, limiting the ability to apply the findings to patients eligible for *RET*-directed therapy [24, 25]. There are several common features observed among patients with *RET* fusions in publications to date, such as the prevalence of non-smoking status and younger age at diagnosis [26–31]. However, there have been other small studies that have not observed significant differences in these factors [32]. Each of these studies included less than 20 patients with *RET* fusion-positive disease. Several larger studies have similarly described patients with *RET* fusions, but no comparison group was available [30, 33–35].

Other studies have tried to estimate the survival outcomes among patients with *RET* fusion-positive NSCLC. Overall survival (OS) and progression-free survival (PFS) estimates in a meta-analysis were based on unadjusted analyses and found no difference in either OS (*n* = 75 patients with *RET* fusions) or PFS (*n* = 24 with *RET* fusions) by *RET* fusion status [36].

The outcomes of these studies remain inconclusive due to small sample [25–32] and due to the lack of a comparison group or failure to adjust for baseline prognostic factors [34–36]. Adjustment for these factors is critical in that multiple prognostic factors are known to differ among patients with *RET* fusions based on these initial descriptive studies (e.g. age, gender, smoking status, line of therapy, treatment received). Because of these issues, there remains uncertainty regarding the outcomes of patients treated with standard therapy who are diagnosed with *RET* fusion-positive NSCLC related to the broader cohort of patients without these fusions.

This observational study was designed to compare the baseline characteristics and clinical outcomes among patients with metastatic NSCLC by *RET* fusion status treated in standard practice settings prior to the approval of selective *RET* inhibitors.

## Methods

### Data source

This retrospective observational study utilized the Flatiron-Foundation Medicine Clinico-Genomics database (CGDB) [37]. The CGDB is a combination of Flatiron Health’s longitudinal database containing electronic health record (EHR) data from over 265 cancer clinics (approximately 800 sites of care) including more than 2 million cancer patients in the US linked to comprehensive genomic profiling data obtained from Foundation Medicine, Inc. (FMI). The de-identified patient-level clinical data from the EHR includes structured data (e.g., laboratory values, prescribed drugs) in addition to unstructured data collected via technology-enabled chart abstraction from physician’s notes and other unstructured documents (e.g., detailed biomarkers). De-identified patient-level genomic data from FMI includes specimen features (e.g., tumor mutation burden, pathologic purity), alteration-level details (e.g., genomic position, reference and alternate alleles, mutant allele count, minor allele frequency), and therapeutic recommendations that were reported to the clinician at the time of testing. Death dates are entered as month and year to protect confidentiality and obtained from three sources: EHR; social security death index; and published obituary notices. All data are de-identified and provisions are in place to prevent re-identification in order to protect patients’ confidentiality. Institutional Review Board (IRB) approval with waiver of informed consent (Copernicus Group IRB) was obtained by Flatiron Health prior to the provision of these datasets and conduct of this study.

### Eligibility criteria

Patients with metastatic NSCLC identified in the CGDB were eligible for this study if they were age 18 years or older at the time of diagnosis, and who received their initial systemic anti-cancer therapy within 180 days of metastatic diagnosis. Patients only treated with adjuvant or neoadjuvant systemic therapy were excluded to avoid the inclusion of patients with early stage disease with missing stage data, but patients who were diagnosed with earlier stage disease who progressed were included if they received systemic therapy within 180 days after progression. All patients in the CGDB had results of next-generation sequencing (NGS) reported in the database from FMI; the sensitivity and specificity to *RET* fusions are both very high with NGS-based methods [38–40] Patients were required to have initiated the systemic therapy on or after January 1, 2011. Patient follow-up data were available through June 2019 for this study, preceding the availability of selective *RET* inhibitors. No minimum follow-up time was required after initiation of first-line therapy.

### Patient cohorts

Patients were assigned to the *RET*+ cohort if there was evidence of a fusion with *RET* recorded at any time in the CGDB. Patients were assigned to the *RET*- cohort if there was no evidence of a *RET* fusion in the database.

### Statistical analyses

Baseline characteristics, defined at start of first-line therapy, were compared between the *RET*+ and *RET*- cohorts using student’s t-test for continuous measures and Chi-squared/Fisher’s exact test for categorical measures. Missing values were reported descriptively and included as a categorical variable in the comparative analyses to avoid losing cases due to missing data. Descriptive analyses were conducted to characterize testing and treatment patterns of both cohorts. Duration of treatment was defined from the start of the line of therapy until the last infusion/administration of any drug within that regimen. Due to the high number of treatment regimens used in NSCLC, multiplicity is a concern and pairwise comparisons of individual treatment patterns were not made but all regimens were reported descriptively.

Analyses were conducted to compare tumor response, PFS and OS between the *RET*+ and *RET*- cohorts. Tumor response outcomes were analysed using Fisher’s exact test, and best response during the line of therapy was categorized as complete response (CR), partial response (PR), stable disease (SD) or progressive disease (PD) as recorded by the oncologist in the patient record. Additionally, odds ratios were calculated for objective response rate (ORR), which combined both CR and PR, and were analysed among patients with response data recorded using multivariable logistic regression by *RET* status. Time-to-event analyses (PFS and OS) were conducted using Kaplan-Meier method and Cox proportional hazards regression from the start of the line of therapy. Baseline covariates in the multivariable regression for the adjusted analysis of PFS and OS included age, sex, race, practice type (academic or community), body weight, body mass index (BMI), stage at initial diagnosis, tumor histology, smoking status, microsatellite instability (MSI) status, genomic alterations, Eastern Cooperative Oncology Group (ECOG) performance status, PD-L1 expression (positive = > 1% staining versus negative), initial treatment regimen (checkpoint inhibitor use yes/no), and reported metastatic sites.

Secondary analyses compared tumor response and evaluated adjusted and unadjusted PFS and OS from the start of first-line therapy among the subgroup of patients in both cohorts who received first-line pembrolizumab + pemetrexed + platinum (pembro + PC, the regimen evaluated in Keynote [KN]-189), which has recently become a standard of care for patients with NSCLC [41]. For the KN-189 regimen analyses, carboplatin and cisplatin were considered interchangeable.

### Sensitivity analyses

Post-hoc sensitivity analyses explored variables reported differentially in the FMI versus EHR datasets (i.e. PD-L1 status). In the CGDB database, PD-L1 is evaluated using immunohistochemistry (IHC) and is assigned categorical groupings so that < 1% staining is negative, 1–49% is low positive, and 50% or greater is considered high positive. In the EHR data, PD-L1 is coded as either positive or negative by abstractors using either percent staining (in the database, any staining > 1% is entered as ‘positive’) or if positivity is explicitly stated in the report in the patient record. Primary analyses combined all data to minimize missingness; sensitivity analyses limited the evaluation of PD-L1 status to FMI test results. Sensitivity analyses also evaluated the influence of several assumptions in the covariates used in the adjusted regression models: 1) excluding missing data; 2) collapsing checkpoint inhibitor use to none, monotherapy or combination therapy regimens; 3) including other targeted agents as a unique drug category and 4) excluding the prior therapy grouping strategy.

## Results

### Patient characteristics

**Fig. 1:**
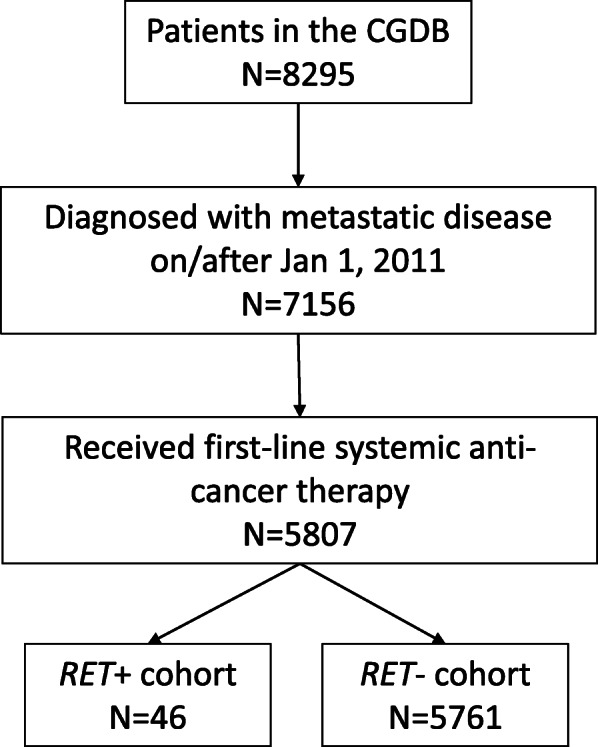
Patient inclusion flow chart

A total of 5807 patients were identified that met the eligibility criteria (*RET*+ cohort, *N* = 46; *RET*- cohort, *N* = 5761). A summary of the cohort identification is included in Fig. 1. Of the overall eligible NSCLC cohort, 46 patients (0.8%) had *RET* fusions.

A summary of the characteristics of patients at the start of first-line therapy (baseline) is provided in Tables 1 and 2. Patients with *RET* fusions were significantly younger (mean age 62.9 versus 67.2 years, *p* = 0.004), were more likely to have non-squamous disease (100% versus 79.4%, *p* = 0.0006), to be non-smokers (63.0% versus 18.1%, *p* < 0.0001), and had better performance status (*p* = 0.02). Of note, no patients with *RET* fusions had ALK, ROS1, KRAS or BRAF positive disease, however, there were three patients with concurrent EGFR mutations where the *RET* fusion was considered to have been acquired due to resistance to ongoing anti-EGFR therapy. Two patients with *RET* fusions had an exon 19 mutation (one with a T790M mutation) and one patient had an exon 21 mutation.

**Table 1:** Demographic characteristics of study cohorts at the start of first-line therapy

Characteristics	Overall ***N*** = 5807	RET+ ***N*** = 46	RET- ***N*** = 5761	RET+ vs RET- ***P*** value^*^
Age, years				
Mean (Sd)	67.2 (10.2)	62.9 (11.0)	67.2 (10.2)	0.004
Median (IQR)	68.1 (60.2, 74.8)	65 (54.8, 70.1)	68.1 (60.3,74.9)	
Sex, n (%)				
Female	2880 (49.6)	23 (50.0)	2857 (49.6)	1.0
Male	2927 (50.4)	23 (50.0)	2904 (50.4)	
Race, n (%)				
White	4078 (76.5)	31 (72.1)	4047 (76.5)	0.76
Asian	187 (3.5)	2 (4.7)	185 (3.5)	
Black	343 (6.4)	3 (7.0)	340 (6.4)	
Other	723 (13.6)	7 (16.3)	716 (13.5)	
Missing/Unknown	476	3	533	
Practice type, n (%)				
Academic	342 (5.9)	4 (8.7)	338 (5.9)	0.35
Community	5465 (94.1)	42 (91.3)	5423 (94.1)	

^*^Missing values are not included in the statistical comparison between cohorts; comparisons based on t-test for continuous variables and Chi square test for categorical variables

*RET* rearranged during transfection; *Sd* standard deviation; *IQR* interquartile range

**Table 2:** Clinical characteristics of study cohorts at the start of first-line therapy

Characteristics	Overall ***N*** = 5807	RET+ ***N*** = 46	RET- ***N*** = 5761	RET+ vs RET- ***P*** value^*^
Body weight (kg)				
Mean (Sd)	75.7 (18.8)	74.9 (16.2)	75.7 (18.8)	0.81
Median (IQR)	73.7 (62.1, 86.4)	70.1 (62.8, 86.4)	73.8 (62.1, 86.4)	
BMI category, n (%)				
Underweight	302 (5.4)	2 (4.7)	300 (5.4)	0.82
Normal	2203 (39.1)	20 (46.5)	2183 (39.0)	
Overweight	1843 (32.7)	13 (30.2)	1830 (32.7)	
Obese	1289 (22.9)	8 (18.6)	1281 (22.9)	
Missing/Unknown	170	3	167	
Stage at initial diagnosis, n (%)				
Stage I	486 (8.5)	3 (6.5)	483 (8.6)	0.34
Stage II	335 (5.9)	0 (0.0)	335 (5.9)	
Stage III	1154 (20.3)	9 (19.6)	1145 (20.3)	
Stage IV	3714 (65.3)	34 (73.9)	3680 (65.2)	
Missing/Unknown	118	0	118	
Histology, n (%)				
Non-squamous	4437 (79.6)	45 (100.0)	4392 (79.4)	< 0.0001
Squamous	1138 (20.4)	0 (0.0)	1138 (20.6)	
Missing/Unknown	232	1	231	
Smoking status, n (%)				
Smoking history	4720 (81.5)	17 (37.0)	4703 (81.9)	< 0.0001
No smoking history	1071 (18.5)	29 (63.0)	1042 (18.1)	
Missing/Unknown	16	0	16	
ECOG performance status, n (%)				
0	1438 (33.8)	19 (61.3)	1419 (33.6)	0.02
1	2088 (49.1)	9 (29.0)	2079 (49.2)	
2	595 (14.0)	2 (6.5)	593 (14.0)	
3+	136 (3.2)	1 (3.2)	135 (3.2)	
Missing/Unknown	1550	15	1535	
*RET* fusion partner				
KIF5B	–	29 (63.0)	–	–
CCDC6	–	11 (23.9)	–	
NCOA4	–	3 (6.5)	–	
Other^d^	–	3 (6.5)	–	
EGFR positive, n (%)				
Yes	447 (18.8)	3 (8.6)	444 (18.9)	0.19
No	1936 (81.2)	32 (91.4)	1904 (81.1)	
Missing/Unknown	3424	11	3413	
ALK positive, n (%)				
Yes	80 (3.7)	0 (0.0)	80 (3.8)	0.64
No	2086 (96.3)	34 (100.0)	2052 (96.3)	
Missing/Unknown	3641	12	3629	
KRAS positive, n (%)				
Yes	233 (24.6)	0 (0.0)	233 (25.0)	0.05
No	714 (75.4)	13 (100.0)	701 (75.1)	
Missing/Unknown	4860	33	4827	
ROS1 positive, n (%)				
Yes	16 (1.2)	0 (0.00)	16 (1.2)	1.0
No	1310 (98.8)	19 (100.0)	1291 (98.8)	
Missing/Unknown	4481	27	4454	
BRAF positive, n (%)				
Yes	42 (4.9)	0 (0.0)	42 (4.9)	1.0
No	820 (95.1)	7 (100.0)	813 (95.1)	
Missing/Unknown	4945	39	4906	
MSI status, n (%)				
MSI high	7 (0.2)	0 (0.0)	7 (0.3)	1.0
MSI stable	2849 (99.3)	22 (100.0)	2827 (99.3)	
MSI intermediate	13 (0.5)	0 (0.0)	13 (0.5)	
Missing/Unknown	2938	24	2914	
PD-L1 expression^a^, n (%)				
Positive (≥ 1%)	1206 (47.4)	15 (68.2)	1191 (47.2)	0.06
Negative	1337 (52.6)	7 (31.8)	1330 (52.8)	
Missing/Unknown	3264	24	3240	
FMI PD-L1 expression^b^, n (%)				
High Positive (≥50%)	390 (31.2)	2 (28.6)	388 (31.2)	0.21
Low Positive (1–49%)	353 (28.2)	4 (57.1)	349 (28.1)	
Negative	507 (40.6)	1 (14.3)	506 (40.7)	
Missing/Unknown	4557	39	4518	
Time from metastatic diagnosis to FMI test^c^, days				
Mean (Sd)	86.7 (427.1)	135.9 (480.4)	86.3 (426.6)	0.43
Median (IQR)	0 (0.0, 49.0)	1.5 (0.0, 441.0)	0 (0.0, 49.0)	
Adrenal metastases, n (%)				
Yes	657 (11.3)	3 (6.5)	654 (11.4)	0.48
Not reported	5150 (88.7)	43 (93.5)	5107 (88.7)	.
Bone metastases, n (%)				
Yes	2018 (34.8)	21 (45.7)	1997 (34.7)	0.12
Not reported	3789 (65.3)	25 (54.4)	3764 (65.3)	
Brain metastases, n (%)				
Yes	1156 (19.9)	9 (19.6)	1147 (19.9)	1.0
Not reported	4651 (80.1)	37 (80.4)	4614 (80.1)	
Distant lymph node metastases, n (%)				
Yes	808 (13.9)	7 (15.2)	801 (13.9)	0.83
Not reported	4999 (86.1)	39 (84.8)	4960 (86.1)	
Liver metastases, n (%)				
Yes	697 (12.0)	13 (28.3)	684 (11.9)	0.0007
Not reported	5110 (88.0)	33 (71.7)	5077 (88.1)	
Other metastases, n (%)				
Yes	4176 (71.9)	27 (58.7)	4149 (72.0)	0.05
Not reported	1631 (28.1)	19 (41.3)	1612 (28.0)	

^*^Missing values are not included in the statistical comparison between cohorts; comparisons based on t-test for continuous variables and Chi square test for categorical variables; Fisher’s exact test was used for cells with frequencies < 5

^a^As reported in the EHR or FMI test results available at the time of initiation of therapy

^b^Limited to FMI test results available at the time of initiation of therapy

^c^Negative days (ie, tests prior to advanced diagnosis) set to zero

^d^Other fusions included TRIM24, GAS2 and FRD4A

*RET* rearranged during transfection; *Sd* standard deviation; *IQR* interquartile range; *BMI* body mass index; *ECOG* Eastern Cooperative Oncology Group; *FMI* Foundation Medicine, Inc.; *EHR* electronic health record; *EGFR* epidermal growth factor receptor; *PD-L1* programmed death ligand 1; *MSI* microsatellite instability; *ALK* anaplastic lymphoma kinase; *KRAS* Kirsten rat sarcoma viral oncogene homolog; *ROS1* ROS proto-oncogene 1; *BRAF* v-raf murine sarcoma viral oncogene homolog B

PD-L1 status as recorded in EHR prior to initiation of first-line therapy was not recorded among 52.2% (*n* = 24) of patients in the *RET*+ cohort and 56.2% (*n* = 3240) of patients in the *RET*- cohort. In the EHR data, patients with PD-L1 positive results prior to initiation of first-line therapy were 68.2 and 47.2%, respectively (*p* = 0.06, Table 2). When limiting the analysis to the IHC-based results from FMI recorded prior to initiation of first-line treatment, patients in the *RET*+ versus *RET*- cohorts were high positive (28.6% versus 31.2%), low positive (57.1% versus 28.1%), and negative (14.3% versus 40.7%), *p* = 0.21. A greater proportion of data were missing (84.8%, *n* = 39 and 78.4%, *n* = 4518 of patients in the *RET*+ and *RET*- cohorts, respectively) when limiting PD-L1 status to only values reported by FMI IHC prior to initiation of therapy.

### Treatment patterns by RET status

**Fig. 2:**
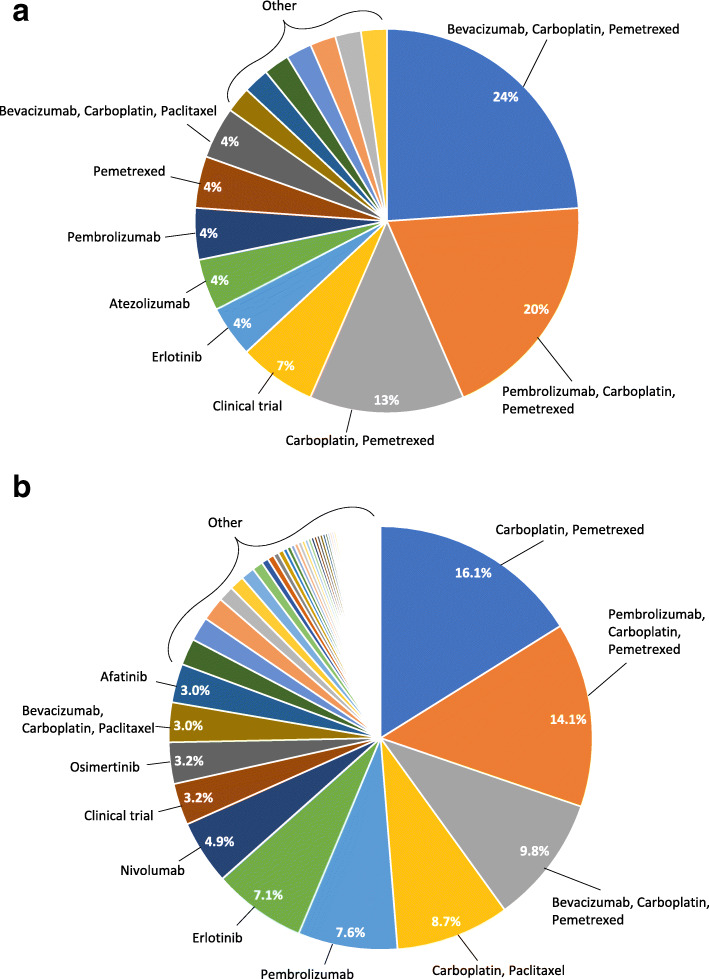
First-line treatment patterns among patients with and without *RET* fusions. A *RET*+ cohort. B *RET*- cohort

The most common regimens used in the first-line setting are presented in Fig. 2 for the *RET*+ (*n* = 46) and *RET*- cohorts (*n* = 4392). The most common first-line regimens used for patients with non-squamous NSCLC were bevacizumab + carboplatin + pemetrexed (23.9% for the *RET*+ and 9.8% for the *RET*- cohort), pembrolizumab + carboplatin + pemetrexed (19.6%, *RET*+; 14.1% *RET*-), pemetrexed + carboplatin (13.0% *RET*+; 16.1% *RET*-). All other regimens were each used in less than 5% of the *RET*+ cohort (other than clinical trial participation, which was 6.5% for *RET*+ and 3.3% of the *RET*- cohort); these other regimens comprised 37.0% of all *RET*+ first-line therapies.

Among patients with *RET*- cancers, carboplatin + paclitaxel was used among 8.7%, pembrolizumab among 7.6%, and erlotinib among 7.1%. The other regimens were each used by less than 5% of the *RET*- cohort and comprised 36.6% of first-line therapies. Overall, 14 patients (30.4%) in the *RET*+ cohort and 1702 (29.5%) in the *RET*- cohort received checkpoint inhibitors in the first line setting. Pembro + PC was used by 9 (19.6%) patients in the *RET*+ cohort and 665 (11.5%) in the *RET*- cohort.

There were no patients with squamous NSCLC identified in the *RET*+ cohort, therefore no summary of treatment patterns by *RET* status could be reported.

The observed median duration of therapy for patients in the *RET*+ cohort receiving pembro + PC was 106 days (range 13–512). The observed median duration of therapy for patients receiving pembro + PC in the *RET*- cohort was 92 days (range 1–748).

### Clinical outcomes

There were no significant differences in tumor response in the first-line setting (*p* = 0.17), for the subgroup who received pembro + PC in the first-line setting (*p* = 0.15) or to second-line therapy (*p* = 0.93) by *RET* fusion status (Table 3). The rate of missing tumor response data was 41.3 and 58.7%, 11.1 and 34.4%, and 39.1 and 49.3% for the *RET*+ and *RET*- cohort, respectively for the first-line, first-line pembro + PC, and second-line settings.

**Table 3:** Tumor response by *RET* fusion status

	RET+ cohort (***N*** = 46)	RET- cohort (***N*** = 5761)	***p***-value^*^
**Any first-line therapy, unadjusted**	**n (%)**^a^	**n (%)**^a^	
Complete response (CR)	1 (3.7)	112 (4.7)	0.17
Partial response (PR)	20 (74.1)	1284 (53.9)	
Stable disease (SD)	2 (7.4)	530 (22.3)	
Progressive Disease (PD)	4 (14.8)	455 (19.1)	
Missing/unknown	19	3380	
**First-line pembro + PC (KN-189 regimen), unadjusted**	***N*** **= 9**	***N*** **= 665**	
CR	1 (12.5)	15 (3.4)	0.15
PR	5 (62.5)	249 (57.1)	
SD	0 (0.0)	100 (22.9)	
PD	2 (25.0)	72 (16.5)	
Missing/unknown	1	229	
**Any second-line therapy, unadjusted**	***N*** **= 23**	***N*** **= 3173**	
CR	0 (0.0)	47 (2.9)	0.93
PR	6 (42.9)	564 (35.1)	
SD	4 (28.6)	478 (29.7)	
PD	4 (28.6)	519 (32.3)	
Missing/unknown	9	1565	
**Objective response rate (ORR)**	**Odds ratio (95% confidence interval)**		***p*-value**
First line, unadjusted, *n* = 2408	2.47 (0.99, 6.14)		0.05
First-line pembro +PC , unadjusted, *n* = 444	1.95 (0.39, 9.80)		0.49
First line, adjusted^b^, *n* = 2408	0.44 (0.17, 1.13)		0.09
First line pembro + PC, adjusted^b^, *n* = 444	0.63 (0.10, 3.84)		0.61
Second line, unadjusted, *n* = 1622	1.22 (0.42, 3.54)		0.78
Second line, adjusted^b^, *n* = 1622	0.58 (0.18, 1.87)		0.36

^*^ Fishers exact test; missing values not included in statistical calculations

^a^Denominator for percentages excludes missing

^b^Mulitvariable adjusted logistic regression

*KN-189* first-line treatment with pembrolizumab (pembro) + pemetrexed + platinum (PC)

As presented in Table 4, unadjusted PFS from the start of first-line therapy was not significantly different by *RET* fusion status (hazard ratio [HR] = 1.40: 95% confidence interval [CI]: 1.0–2.0, *p* = 0.06). There was a significant difference favoring longer OS for the *RET*+ cohort in unadjusted analyses (HR = 1.91: 95% CI: 1.2–3.0, *p* = 0.005). Unadjusted PFS and OS by *RET* status were not significantly different for patients receiving first-line KN-189 regimen (PFS HR = 1.0; 95% CI: 0.5–2.3, *p* = 1.0; OS HR = 1.9; 95% CI: 0.48–7.72, *p* = 0.36). Among those who received pembro + PC, median PFS for the *RET*+ cohort was 6.6 months (95% CI: 0.4 - not reached) and was 5.7 months (95% CI: 5.0–6.5) for those without *RET* fusions. Median Table OS was not reached for patients in the *RET*+ cohort receiving the KN-189 regimen and the CI could not be evaluated but was 14.0 months (95% CI: 12.3–18.5) for patients without *RET* fusions.

**Table 4:** Progression-free and overall survival of *RET*+ versus *RET*- cohorts from start of first-line therapy

Primary analysis, adjusted for covariates at the start of first line therapy			
Outcome	N	Hazard Ratio, HR (95% confidence interval, CI)	***P***-value^*^
**Adjusted analyses**			
Progression-free survival (PFS)	5807	1.24 (0.86, 1.78)	0.25
Overall survival (OS)	5807	1.52 (0.95, 2.43)	0.08
PFS, Pembro + PC (KN-189 regimen)	674	1.07 (0.44, 2.61)	0.89
OS, Pembro + PC	674	1.54 (0.36–6.62)	0.56
**Unadjusted analyses**			
PFS	5807	1.40 (0.99–2.00)	0.06
OS	5807	1.91 (1.22–3.00)	0.005
PFS, Pembro + PC	5807	1.01 (0.45–2.27)	0.98
OS, Pembro +PC	5807	1.92 (0.48–7.72)	0.36
**Sensitivity analyses**			
PFS^a^	5807	1.23 (0.86–1.78)	0.26
OS^a^	5807	1.53 (0.96–2.44)	0.08
PFS^b^	5807	1.27 (0.88–1.84)	0.20
OS^b^	5807	1.56 (0.98–2.50)	0.06
PFS^c^	5807	1.24 (0.86–1.78)	0.26
OS^c^	5807	1.53 (0.95–2.44)	0.08
PFS^d^	3615	1.10 (0.67–1.82)	0.71
OS^d^	3615	1.47 (0.78–2.75)	0.23

^*^ Multivariable regression, adjusted for age, sex, race, practice type (academic or community), body weight, body mass index (BMI), stage at initial diagnosis, tumor histology, smoking status, microsatellite instability (MSI) status, genomic alterations, Eastern Cooperative Oncology Group (ECOG) performance status, PD-L1 expression (positive = > 1% staining versus negative), initial treatment regimen, and reported metastatic sites

^a^Covariate of therapy received: Checkpoint inhibitor (ICI) monotherapy vs ICI combination therapy vs other treatments

^b^Covariate of therapy received Checkpoint inhibitor (ICI) monotherapy vs ICI combination therapy vs biologic therapy vs other treatments

^c^Not including therapy received as a covariate

^d^Excluding missing variables

*KN-189* first-line treatment with pembrolizumab (pembro) + pemetrexed + platinum (PC)

After adjusting for all covariates, no statistically significant differences remained for either PFS (HR = 1.24, 95% CI:0.86–1.78, *p* = 0.25) or OS (HR = 1.52, 95% CI: 0.95–2.43, *p* = 0.08) outcomes between the *RET*+ and *RET*- cohorts (Table 4).

### Sensitivity analyses

When further categorizing drug class as checkpoint inhibitor-based monotherapy versus checkpoint inhibitor-based combination therapy, versus all others, this factor (drug class of first-line therapy) remained similar between *RET* cohorts (*p* = 0.78 and *p* = 0.07 in the PFS and OS analyses, respectively), and did not alter significance of the primary findings for PFS (HR = 1.23, 95% CI: 0.86–1.78, *p* = 0.26) or OS (HR = 1.53, 95% CI: 0.96–2.44, *p* = 0.08) by *RET* status. Similarly, further categorization within treatments received to include targeted or biologic agents as an additional group (PFS HR = 1.27, 95% CI: 0.88–1.84, *p* = 0.20; OS HR = 1.56, 95% CI: 0.98–2.5, *p* = 0.06) or by excluding prior treatment as a covariate (PFS HR = 1.24, 95% CI: 0.86–1.78, *p* = 0.26; OS HR = 1.53, 95% CI: 0.95–2.44, *p* = 0.08) did not alter any of the results. Lastly, the exclusion of missing values in the analysis also did not change the results (PFS HR = 1.10, 95% CI: 067–1.82, *p* = 0.71; OS HR = 1.47; 95% CI: 0.78–2.75, *p* = 0.23), Table 4.

## Discussion

This large real-world database study of over 5900 patients with NSCLC treated in the US identified 46 (0.8%) patients with a *RET* fusion. The observed *RET* fusion rate in this study is comparable to other cohorts of patients, which range from 0.6–1.8% [24, 27, 32, 33, 42, 43]. A number of other studies were limited to patients without other actionable alterations and reported higher prevalence in these cohorts, ranging from 2.2–3.8%, potentially due to the much smaller denominator [25, 28, 44, 45]. Additionally, the inclusion of both patients with and without *RET* fusions from the same database allows for comparative analyses to be conducted to more accurately characterize differences observed in standard practice with and without this alteration.

There have been inconsistent findings related to differences in demographic and clinical characteristics between patients with and without *RET* fusions [25, 28, 32, 36]. Patients with *RET* fusions in this study were younger (*p* = 0.004), more likely to be diagnosed with non-squamous cell carcinoma (*p* < 0.001), nonsmokers (*p* < 0.0001) and had better performance status (*p* = 0.01). These findings have been observed previously [24, 26, 36]. However, in this study, stage at diagnosis and sex were not significantly different by *RET* status. The Flatiron NSCLC database is limited to patients with advanced or metastatic disease; therefore, patients diagnosed with early stage disease who did not progress to a metastatic stage would not be included. Therefore, early stage disease cannot fully be evaluated in this dataset. Differences in sex have also been observed in some studies [24, 28, 36]. This study did not find any differences in sex, but this could also be due to the restriction of the cohort to those patients whose disease had advanced, or due to the non-comparative design of previous studies, which may limit their interpretation [27, 28, 46].

In this study, PD-L1 was used as a covariate for the comparative analyses and limited to data that were recorded prior to initiation of first-line therapy. Therefore, these data are not intended to compare PD-L1 status by *RET* status, given the exclusion of any variables recorded after treatment initiation. This study demonstrated 68.2% positivity in the EHR and 85.7% in the FMI report for the tests conducted prior to initiation of first-line therapy. Additional work would be needed taking all PD-L1 tests into consideration to evaluate expression levels by *RET* status.

In the Flatiron Health EHR data record, the presence of metastatic sites is not a fully reliable field as only presence of metastases are recorded. The absence of a metastatic site does not mean that the patient did not have metastases. A chart review study would be needed to better identify metastatic sites and was not the intent to investigate these differences by cohort, but rather to characterize baseline data for covariate adjustment in the outcomes analysis. Therefore, these data should be interpreted with caution.

Patients with and without *RET* fusions receiving standard non-*RET* targeted therapies had similar PFS and OS outcomes after adjusting for baseline factors; these similarities were maintained when limited to the KN-189 regimen and when excluding missing variables in sensitivity analysis. This suggests that patients with *RET* fusions have similar outcomes on standard therapy as their fusion-negative counterparts when adjusting for demographic, clinical and treatment-related factors. While treatment patterns were too heterogeneous to adjust for every regimen, the grouping of treatment classes by three methods made little difference in clinical outcomes. As expected, the inclusion of targeted or biologic therapies was meaningful for improved patient outcomes, but it did not alter the significance of findings in the comparison by *RET* status. There were no specific regimen differences between the cohorts that might otherwise explain potentially unadjusted factors to explain survival outcomes. Of note, this study included an unselected cohort of patients with NSCLC. Several factors were notable, such as the lack of patients with squamous cell carcinoma or co-existing mutations. The single-arm trial, LIBRETTO-001 reported the outcomes of 105 patients with RET-fusion positive NSCLC. In this study, one patient had squamous cell carcinoma [18]. The current study supports the rarity of *RET* fusions in squamous NSCLC. Future research may wish to evaluate a more selected cohort for comparison, whereas in this study we focused on all potential differences by cohort, including histology, and then adjusted for those covariates in comparative analyses. The purpose of this study was not to compare to the clinical trial, but rather suggests that real-world data may provide such a data source for an external control arm in future research.

One of the limitations of real-world data is the type of variables recorded. There may be additional important factors that this analysis could not account for due to the lack of certain prognostic variables in EHR. Comorbid conditions, for example, are not available for analysis in the Flatiron datasets, and patient well-being can only be estimated through their reported performance status. Comorbidities are known to have a prognostic effect on clinical outcomes in oncology [47]. Assuming that patients with better performance status and lower age may have fewer comorbidities, the inclusion of comorbid conditions may have further reduced any potential differences by *RET* status, but this remains unknown and should be further investigated as larger datasets become available that include these data. Additionally, clinical variables such as tumor grade and details on the histological subtypes of non-squamous disease are also lacking in this real-world dataset that could further inform this analysis. While the results were consistent across all subgroups and analyses in this study, the results are limited by the small sample size, particularly for KN-189, and should be further evaluated as testing rates improve and greater numbers of patients with *RET* fusions become available for study.

The Flatiron CGDB is not a representative sample but is limited to practices in the network and to patients with FMI test results. This cohort is known to have immortal time bias, so the survival estimates from this study will be longer than those in a general population [48]. Since both the *RET*+ and *RET*- cohorts experience this issue, the comparative analyses are not impacted by this bias in the current study, but the duration of overall survival outcomes of these cohorts are expected to be longer than what may be observed outside of this dataset. These findings are likely not generalizable to the overall US population.

Due to the rarity of *RET* fusions among patients with NSCLC, the robust study of the prognostic effect of these fusions remains challenging. It will be important that genomic testing become more widespread to identify fusions and that this factor be subsequently recorded in EHR datasets to enable a more robust study. This study was limited to patients in Flatiron’s CGDB in order to identify *RET* fusions. There were 8295 patients in this dataset from which eligibility criteria were applied. In the EHR, there are longitudinal data from more than 60,000 patients with advanced or metastatic disease available for study. As *RET* becomes hard coded into EHR systems, researchers will not have to rely on the linkage to genomics testing results for patient identification and will be able to access larger cohorts directly from EHR data for observational research.

## Conclusion

This cohort study evaluated characteristics, treatment patterns and compared clinical outcomes among patients with NSCLC by *RET* fusion status. The findings confirm the differential characteristics of patients with *RET* fusions, that contribute to favorable clinical outcomes in the setting of standard non-*RET* targeted therapies. However, after adjusting for baseline covariates, there remains little evidence that the *RET* fusion alone contributes to these outcomes. Therapies targeting the *RET* fusion as a driver in NSCLC may help improve outcomes in this patient population. This study is limited due to small sample size and potential unmeasured confounding and was not designed to evaluate the prognostic effect of the presence of a *RET* fusion in NSCLC.

## Data Availability

The data that support the findings of this study have been originated by Flatiron Health, Inc. These de-identified data may be made available upon request and are subject to a license agreement with Flatiron Health; interested researchers should contact <DataAccess@flatiron.com> to determine licensing terms.

## Abbreviations

ALK: Anaplastic lymphoma kinase
BMI: Body mass index
BRAF: V-raf murine sarcoma viral oncogene homolog B
CI: Confidence interval
CR: Complete response
CGDB: Clinico-Genomics database
ECOG: Eastern Cooperative Oncology Group
EGFR: Epidermal growth factor receptor
EHR: Electronic health record
FISH: Fluorescent in situ hybridization
FMI: Foundation Medicine, Inc.
HR: Hazard ratio
IQR: Interquartile range
KN-189: Keynote 189
KRAS: Kirsten rat sarcoma viral oncogene homolog
MSI: Microsatellite instability
MET: Mesenchymal-epithelial transition/MET proto-oncogene
NGS: Next generation sequencing
NSCLC: Non-small cell lung cancer
NTRK: Neurotrophic tyrosine receptor kinase
ORR: Objective response rate
OS: Overall survival
Pembro+PC: Pembrolizumab + pemetrexed + platinum
PD: Progressive disease
PD-L1: Programmed death ligand 1
PFS: Progression-free survival
PR: Partial response
RET: Rearranged during transfection
ROS1: ROS proto-oncogene 1
Sd: Standard deviation
SD: Stable disease
TTD: Time to treatment discontinuation
US: United States
