# Correction: Characteristics and outcomes of patients with *RET*-fusion positive non-small lung cancer in real-world practice in the United States

**DOI:** 10.1186/s12885-023-10879-2

**Published:** 2023-04-26

**Authors:** Lisa M. Hess, Yimei Han, Yajun Emily Zhu, Naleen Raj Bhandari, Anthony Sireci

**Affiliations:** 1 Eli Lilly and Company, Indianapolis, IN 46254 USA; 2 Loxo Oncology at Lilly, a wholly owned subsidiary of Eli Lilly and Company, Stamford, CT USA

**Correction:** ***BMC Cancer*** **21, 28 (2021)**

10.1186/s12885-020-07714-3

Following publication of the original article [1], the authors identified an error in the caption of table 4.

In the published article [1], the title of Table 4 is “Progression-free and overall survival of RET + versus RET- cohorts from start of first-line therapy,” which should be corrected to read “Progression-free and overall survival of **RET-** versus **RET +** cohorts from start of first-line therapy,” (correction in bold) to correctly indicate the direction of the hazard ratios.
